# *Sporosarcina pasteurii* can be used to print a layer of calcium carbonate

**DOI:** 10.1002/elsc.202100074

**Published:** 2022-06-16

**Authors:** Niklas Erdmann, Felix Kästner, Kristin de Payrebrune, Dorina Strieth

**Affiliations:** ^1^ Chair of Bioprocess Engineering Technical University of Kaiserslautern Kaiserslautern Germany; ^2^ Chair for Computional Physics in Engineering Technical University of Kaiserslautern Kaiserslautern Germany

**Keywords:** 3D printing, microbially induced calcium carbonate precipitation (MICP), *Sporosarcina pasteurii*, ureolytic activity

## Abstract

When using microbiologically induced calcium carbonate precipitation (MICP) to produce calcium carbonate crystals in the cavities between mineral particles to consolidate them, the inhomogeneous distribution of the precipitated calcium carbonate poses a problem for the production of construction materials with consistent parameters. Various approaches have been investigated in the literature to increase the homogeneity of consolidated samples. One approach can be the targeted application of ureolytic organisms by 3D printing. However, to date, this possibility has been little explored in the literature. In this study, the potential to use MICP to print calcium carbonate layers on mineral particles will be investigated. For this purpose, a dispensing unit was modified to apply both a suspension of *Sporosarcina pasteurii* and a calcination solution containing urea and calcium chloride onto quartz sand. The study showed that after passing through the nozzle, *S. pasteurii* preserved consistent cell vitality and therefore its potential of MICP. Applying cell suspension and calcination solution through a printing nozzle resulted in a layer of calcium carbonate crystals on quartz sand. This observation demonstrated the proof of concept of printing calcium carbonate by MICP through the nozzle of a dispensing unit. Furthermore, it was shown that cell suspensions of *S. pasteurii* can be stored at 4°C for a period of 17 days while maintaining its optical density, urease activity and cell vitality and therefore the potential for MICP. This initial concept could be extended in further research to printing three‐dimensional (3D) objects to solve the problem of homogeneity in consolidated mineral particles.

**Abbreviation**
MICP: microbially induced calcium carbonate precipitation

## 1 INTRODUCTION

The production of concrete, the most widely used building material worldwide, accounts for 8.6% of anthropogenic CO_2_ emissions [1]. The reason for this is the high energy requirement for the production of cement, the main binding agent of concrete since cement is produced from limestone at a temperature of 1450°C [2]. Various microorganisms can produce calcium carbonate. This process is called microbiologically induced calcium carbonate precipitation (MICP). The calcium carbonate formed in this process can form bridges between mineral particles, similar to classical binders such as cement, and thus consolidate them. Since the optimal temperature for MICP is 20–50°C, MICP has the potential to produce a building material with a lower energy demand. Past studies have already shown that MICP can be used to increase strength [3, 4] and repair cracks in concrete and sandstone [5, 6]. The production of novel building materials by MICP has also been described in various studies [7, 8, 9, 10]. The mechanism most commonly used for this purpose is ureolysis [11, 12]. This mechanism is easy to control and the necessary reagents are readily available [13]. During ureolytic hydrolysis, one mole of urea is hydrolyzed by urease (EC 3.5.1.5) [14] into two moles of ammonia and one mole of carbonic acid (Equations 1 and 2). Both products hydrolyze (Equations 3 and 4) forming carbonate ions and increasing the pH. In the presence of soluble calcium ions, the carbonate ions formed are precipitated as calcium carbonate (Equation 5).

(1) $$CO{\left(NH_{2}\right)}_{2}+H_{2}O\to NH_{2}COOH+NH_{3}$$

(2) $$NH_{2}COOH+H_{2}O\to H_{2}CO_{3}+NH_{3}$$

(3) $$2NH_{3}+2H_{2}O↔2NH^{4+}+2OH^{-}$$

(4) $$2OH^{-}+H_{2}CO_{3}↔CO_{3}^{2-}+2H_{2}O$$

(5) $$Ca^{2+}+CO_{3}^{2-}\to CaCO_{3}$$

(6) $$CO{\left(NH_{2}\right)}_{2}+2\;H_{2}O+Ca^{2+}\to 2NH_{4}^{+}+CaCO_{3}$$

**PRACTICAL APPLICATION**

Microbiologically induced calcium carbonate precipitation (MICP) has the potential to produce an ecological alternative to conventional cement in building materials through the formation of calcium carbonate. These materials often lack homogeneity of carbonate in the produced samples and therefore consolidation is poor. We demonstrate that printing of a calcium carbonate layer utilizing MICP is possible. We demonstrate that printing of calcium carbonate layers utilizing MICP is possible. In further research this concept can be expanded to achieve true 3D printing with MICP. *Sporosarcina pasteurii* is able to maintain its ability to induce calcium carbonate precipitation during storage at low temperatures as well as during its passage through the printer nozzle. The application of this technology in 3D printing processes could offer the potential to address the issue of unevenly consolidated samples. It also offers the potential to provide artificial sandstone in suitable shapes for the restoration of historic sandstone, for example.

An important factor for the formation of calcium carbonate is the presence of nucleation sites, which serve as initial nucleation cells for the formation of calcium carbonate ions [11, 12]. The negatively charged cell walls of the microorganisms play a supporting role in this process. Positively charged calcium ions in the environment of the microorganisms are attracted to carboxy and phosphoryl groups on the cell surface by electrostatic interactions [15, 16, 17]. This increases the calcium concentration near the cells. Calcium carbonate precipitation subsequently occurs at this nucleation site in the liquid phase around the cell. The influence of cells as nucleation sites is also reflected in the fact that MICP is more efficient compared to enzymatically induced calcium carbonate precipitation (EICP). For example, Zhao et al. showed that when free urease was used instead of *S. pasteurii*, a lower amount of calcium carbonate was precipitated from a solution of urea and calcium chloride. In addition, samples treated by EICP had a lower unconfined compressive strength in relation to the calcium carbonate content than samples treated by MICP [18].To consolidate mineral particles using ureolytic organisms, various methods have been used in the literature. The three main components of these processes are mineral particles, cell suspension of ureolytic microorganisms, for example *S. pasteurii, Bacillus megaterium*, or *Lysinibacillus sphaericus*, and a calcination solution containing urea and a calcium source. Since calcium carbonate precipitation starts immediately after mixing of cell suspension and calcination solution, mixing of the components before application to the sand leads to clogging of the pores in the upper part of the samples, resulting in an inhomogeneous distribution of calcium carbonate in the samples. Various methods have been investigated to circumvent this effect. A commonly used method to consolidate sand is two‐phase injection. After the addition of cell suspension, a curing time is applied to achieve a better fixation of the cells on the sand [19, 20]. This reduced the accumulation of calcium carbonate at the injection point and achieved a more uniform distribution of calcium carbonate. However, the distribution of calcium carbonate in samples produced with two‐phase injection is not completely homogeneous [21]. Another way to increase the homogeneity of the samples is to use a one‐phase injection. To avoid direct precipitation of calcium carbonate during injection, a lag‐phase was achieved by Cheng et al. [22] by adjusting the biomass concentration, urease activity, and pH after mixing the components, which delays the begin of calcium carbonate precipitation [22]. Since in these methods the distribution of the cell suspension is achieved by flushing the sample, there is a filtering effect by the particles, which causes the cells to be distributed unevenly in the sample [23]. Furthermore, these methods result in non‐uniform transport of calcination reagents [24]. This makes it difficult to obtain a homogeneous distribution of calcium carbonate crystals. A more uniform distribution of calcium carbonate in the samples was achieved by Zhao et al. [18] using a method of immersion. For this purpose, sand was first mixed with cell suspension, which initially distributed the cells evenly in the sample. Then, this mixture was placed in geotextile molds and immersed in the calcination solution. The high number of pores allows nutrients to pass through the geotextile into the sample, where the MICP occurs, and the samples are consolidated. A uniform distribution of microorganisms was achieved by Cheng et al. [24] by developing a so‐called bioslurry. For this purpose, ureolytic organisms were mixed with a urea and calcium source after cultivation, causing the cells to encapsulate in calcium carbonate. The precipitated calcium carbonate was then harvested and lyophilized. This resulted in a powder with ureolytic activity. By mixing this powder with sand a homogeneous distribution of the cells in the sand can be achieved [24]. The samples were then consolidated by injecting a calcination solution. Through this method, Cheng et al. [24] were able to achieve a nearly homogeneous distribution of calcium carbonate along a 275 mm long sample. Thus, the immersion and bioslurry methods can solve the problem of sample homogeneity. However, the strength of the samples in both methods is limited by the initial number of cells in the sample. Once all cells are encapsulated, no further consolidation of the samples is achieved. A possible approach to provide both a homogeneous distribution of the calcination solution and a sufficient number of cells and thus nucleation sites could be 3D printing. In fact, the method of bioslurry and the method of immersion have already been combined by Nething et al. [25] to enable a 3D printing process by powder printing. For this purpose, silica sand was mixed with bioslurry in the areas that would later be consolidated, while the outer bed consisted of untreated sand. After printing these layers, the printed bed was immersed in a calcination solution, thus consolidating the structure. In this study, the possibilities of printing cell suspension and cementation solution directly on a surface for MICP are explored. For this purpose, it was first investigated whether *S. pasteurii* can survive passing through different nozzle sizes of a dispensing system. In addition, it was investigated how long a cell suspension of *S. pasteurii* can maintain its potential for MICP during sterile storage at different temperatures. Subsequently, as a proof of concept, a layer of quartz sand was consolidated using MICP. If it is possible to print one layer of calcium carbonate on mineral particles the findings could lead to the development of a 3D printer that is able to produce samples with uniform consolidation without the drawbacks of a fixed number of cells in the sample or the necessity of an immersion of the sample in calcination solution.

## 2 MATERIALS AND METHODS

### 2.1 Cultivation method

*S. pasteurii* (ATTC 11859) was used as ureolytic microorganism as this strain has a very high specific ureolytic activity, its urease synthesis is not repressed by ammonium, has no known pathogenicity [26] and is therefore the most common used strain for MICP. The strain was cultured in NH4‐YE medium as recommended by ATTC. The medium contained 15.75 g Tris buffer (Carl Roth GmbH), 10 g ammonium sulfate (Sigma Aldrich) and 20 g yeast extract (Carl Roth GmbH) per liter. For preparation of the medium, Tris buffer was adjusted to pH 9.2 and divided into two equal parts. Yeast extract and ammonium sulfate were dissolved separately each in one part. The solutions were autoclaved at 121°C for 20 min. After cooling, the solutions were combined under sterile conditions to obtain the final medium. For cultivation, 200 ml of NH4‐YE medium was placed in 500 ml Erlenmeyer flasks and inoculated with 1 v/v % of a pre‐culture that was grown overnight. Cultivation was performed at 120 rpm at 30°C. During the cultivation samples were taken under sterile conditions and the optical density (OD_600_) was measured at a wavelength of 600 nm (Cary 60 UV‐Vis, Agilent Technologies, USA).

### 2.2 Measurement of urease activity

Urease activity was measured using the conductivity method [7]. For this purpose, 1 ml of cell suspension was added to 19 ml of a solution containing a concentration of 1.053 M urea and 10 mM Tris buffer. The change in conductivity was recorded over 5 min. To correlate the change in conductivity with the amount of urea degraded, urea standards were completely hydrolyzed with urease (50–250 mM) from Jackbean (Carl Roth GmbH) and the change in conductivity after complete degradation was measured.

### 2.3 Measurement of cell vitality

Cell vitality was determined by resazurin assay according to the method of Mehring et al. [27]. For this purpose, the cell suspension was diluted to an OD of 0.2 with Sorensen buffer (pH 7.4). For each 20 μl of cell suspension, another 180 μl of Sorensen buffer and 20 μl of a resazurin solution (2 mg/ml) (Sigma Aldrich) were added to a 96 well plate. Fluorescence was measured at 540 nm excitation and 590 nm emission (2030 Multilable Reader VICTOR X3, PerkinElmer, USA). Calibration was performed using standards that contained 0%–100% live cells of *S. pasteurii*.

### 2.4 Storage of cell suspension

To investigate storage stability, a culture of *S. pasteurii* was harvested in exponential phase (OD 2.0) and transferred as aliquots to sterile 2 ml reaction tubes. The aliquots were stored in a drying oven (25°C), in a refrigerator (4°C) and in a freezer (–18°C) to obtain different storage conditions. The optical density, urease activity, and cell viability of the stored aliquots were checked at regular intervals.

### 2.5 MICP printer

**FIGURE 1:**
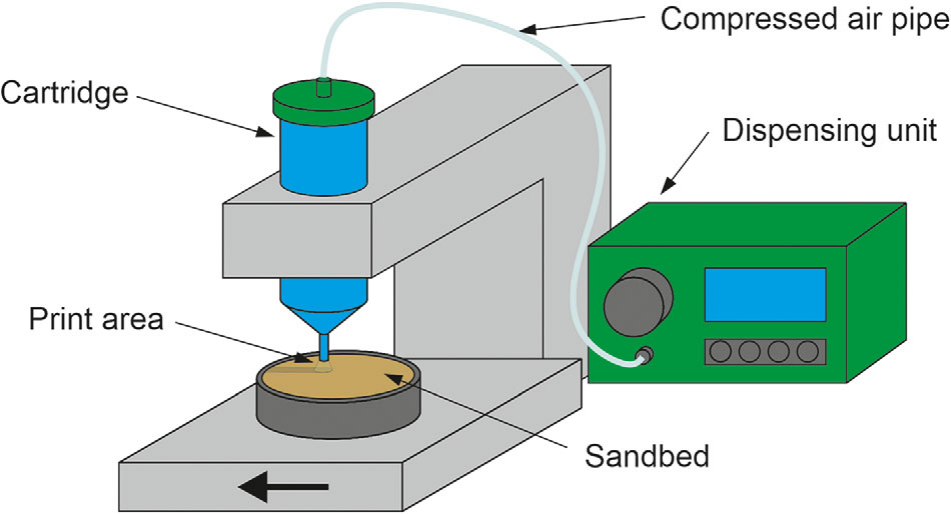
Prototype printer based on an dispensing unit for applying the cell suspension and the calcination solution

A printer (see Figure 1) based on a dispensing unit (DC 1000 series, Vieweg) was used to apply the cell suspension and the calcination solution. The use of this dispensing unit makes it possible to use a variety of different dispensing tips for the application of the respective solutions. This makes it possible to test different flow rates and identify the optimal configuration for printing the calcium carbonate layer. Initial tests were carried out with dispensing tips with diameters of 150, 200, and 250 μm. The device is pressure driven and allows the use of pressure up to 2.76 bar. In order to print directly on a sand bed, it has been necessary to build a printer that can accommodate the cartridge with the dispensing tip. To provide a prototype that can be easily modified and quickly implemented, this is made from interlocking bricks (Lego Technic, The Lego Group). With this motor‐controlled prototype the sand bed can be moved the cartridge while applying the respective solution. For the time being, the printer is designed to reliably print wide bars on sand in one layer.

### 2.6 MICP treatment

A calcination solution of urea (Carl Roth GmbH) and calcium chloride of various concentrations (Carl Roth GmbH) was prepared for MICP. The cell suspension of *S. pasteurii* was harvested after cultivation to an optical density of 2.0 and stored sterile at 4°C. Quartz sand from Haltern, Germany with a quartz content of 98 % was used for this study. Particle sizes ranged from 125 μm to 500 μm with the largest percentage of particles (43%) having a size between 125 and 250 μm. For consolidation of silica sand, cell suspension was first added by the printer to the sand through a nozzle with a diameter of 200 μm under a pressure of 0.69 bar. For the negative control NH4‐YE medium was added instead of cell suspension under the same conditions. Then, after 15 min, an equivalent volume of calcination solution was added. This was followed by a period of 3.5 h during which the calcination solution used was allowed to react. Before each cycle the solution was washed with deionized water to wash off excess salts from the previous cycle. Between the intervals the container was closed with a layer of plastic film to prevent the sample from drying out. Following the sixth printing interval, the sand bed was oven dried for 48 h at 55°C. Subsequently, the sand bed was treated with Resazurin Red S for staining the calcium carbonate.

### 2.7 Staining of calcium carbonate

Alizarin Red S was used to stain calcium carbonate. The yellow dye changes to red in the presence of calcium carbonate. Total 10 mg Alizarin Red S (Carl Roth GmbH) was dissolved in 100 ml 0.2% HCl (Carl Roth GmbH). The solution was sprayed with a spray flask on the entire sand bed after printing.

### 2.8 Microscopic imaging

Microscopic images were taken using a digital reflected light microscope (Digital microscope VHX 7000, KEYENCE, Japan). Images were taken with bright field illumination and a polarizing filter to reduce reflective effects.

## 3 RESULTS AND DISCUSSION

### 3.1 Storage stability of *S. pasteurii*

**FIGURE 2:**
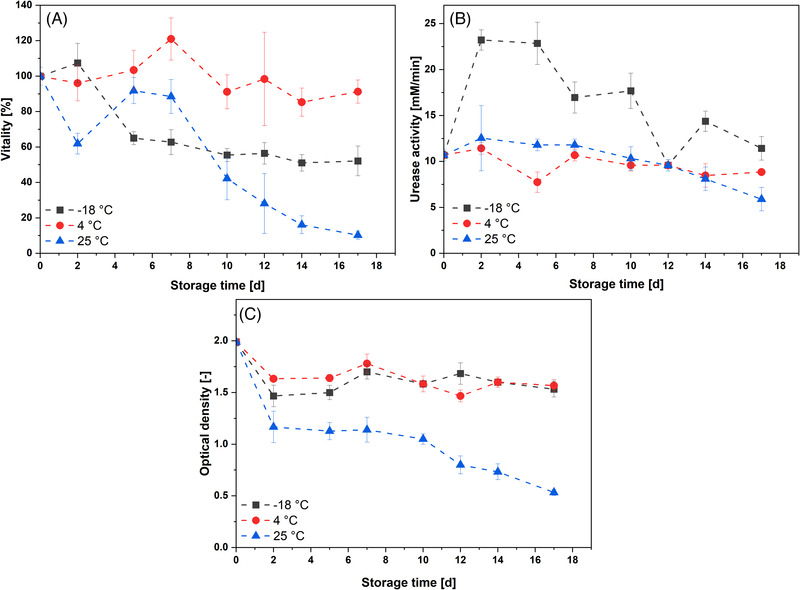
Storage stability of *S. pasteurii* in sterile reaction vials at –18°C, 4°C, and 25°C. A) cell vitality B) urease activity, C) OD_600_. The error bars represent the ± standard deviation for *n* = 3 replicates

To obtain uniform results during MICP the cell suspension used during each cycle of MICP should have similar cell density, urease activity and cell vitality. In order to determine for which period of time a cell suspension of *S. pasteurii* can be used for MICP, the cell suspension was investigated with regard to these parameters. This should give an indication of how long the cells can be stored under different conditions after fermentation while maintaining their potential for MICP. For this purpose, *S. pasteurii* was harvested in the exponential growth phase and stored sterilely as aliquots at various temperatures (–18°C, 4°C, and 25°C). Over the storage period, the cell density initially shows a decrease for all temperatures, presumably as a shock response (see Figure 2C). Subsequently, the cell densities remain constant at about OD_600_ 1.5 over a period of 16 days when stored at –18°C and 4°C. At a storage temperature of 25°C, the cell density drops constantly to a value of OD_600_ 0.53 ± 0.02. The decrease in cell density and thus nucleation sites may ensure that the formation of new crystals at nucleation sites decreases, increasing the growth of pre‐existing calcium carbonate crystals, leading to the formation of larger calcium carbonate crystals [28, 29]. Since larger calcium carbonate crystals allow higher consolidation of silica sand than smaller crystals [30], a decrease in cell density can thus lead to higher calcination efficiency. At the same time, a sufficient amount of biomass and thus nucleation sites must be present to allow the growth of new crystals. A strong decrease of the biomass concentration could lead to a decrease of the calcination efficiency. Storage at –18°C and 4°C thus seems uncritical with regard to cell density, while storage at 25°C could lead to problems with MICP. However, the function of the cells as nucleation sites depends, among other things, on whether the cell excretes ammonium ions to obtain ATP [26]. An indicator of whether cells are maintaining their metabolic processes can be obtained by measuring cell vitality. While the cell vitality remains almost constant over the storage period at 4°C, the vitality decreases to 52.2 ± 8.4% within 17 days when stored at –18°C, while at 25°C the vitality decreases to 10.3 ± 2.3% (see Figure 2A). The decrease in vitality during storage at 25°C is most likely due to the fact that the cells continue to grow at this temperature and enter the death phase, which is also reflected in the decrease in cell density. The decrease in cell viability during storage at –18°C, on the other hand, can be attributed to damage to the cells due to freeze‐thaw stress, since no protective agent against ice crystal formation was added for storage. In addition to their function as nucleation sites, the cells need to maintain their ureolytic activity for MICP. Storage at –18°C shows a strong increase in ureolytic activity in the first days of storage (see Figure 2B). Since urease from *S. pasteurii* is intracellularly, this increase is most likely due to damage to the cells during freezing, whereby urease is released from the cells and is present as free urease which declines in its activity over the following storage time. Since free urease is not limited by mass transport of the substrate urea into the cell, the observed urease activity increases. During storage at 4°C and 25°C, the urease appears to remain present intracellularly and is thus protected from loss of activity. The findings of Bachmeier et al. [31] support this thesis. They describe that free urease isolated from *S. pasteurii*, dropped to an activity of 10% within a few days of storage at 30°C. In a similar experiment, Konstantinou et al. [32] found that the urease activity of a culture of *S. pasteurii* which was grown in NH4‐YE medium increased after day 14 of storage at 4°C. They attribute this increase to a slow growth of *S. pasteurii* during storage. However, in the study by Konstantinou et al. [32] the initial cell density during the experiment was OD_600_ 1.0. This suggests that the cells have grown less dense and therefore consumed a different amount of nutrients which might lead to different results in the stability of the urease activity. The results of cell density, vitality and ureolytic activity suggest that it is appropriate to store cells of *S. pasteurii* at –18°C or 4°C for a period of 17 days and use them for subsequent MICP. Storage at 25°C should be avoided, as the vitality of the cells and thus their function as nucleation sites is not guaranteed. For use in a printer, however, storage at 4°C would be more appropriate, as the cell suspension can then be applied directly from a cooled storage vessel via a nozzle, whereas a frozen suspension would first have to be thawed.

### 3.2 Stability of cells through printing nozzle

**FIGURE 3:**
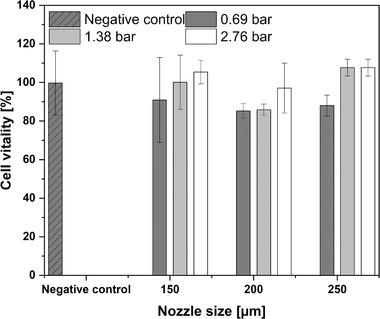
Comparison of cell vitality of *S. pasteurii* before and after passing through nozzles of various sizes (150–250 μm) at different pressure levels (0.69–2.76 bar). The error bars represent the ± standard deviation for *n* = 5 replicates

Since the printer is based on a dispensing unit that is operated with pressure it is necessary to evaluate if *S. pasteurii* cells can pass through different nozzles unharmed. For this purpose, cell vitality was investigated. Nozzle sizes of 150, 200, and 250 μm were chosen, while three pressure levels were tested (0.69, 1.38, and 2.76 bar). The Mann‐Whitney U test was performed to determine whether the cell vitality drops significantly after passing through the printing nozzle. Null hypothesis: The difference in the position under the sample is zero. If the *p* value was higher than 0.05, the hypothesis was discarded. No parameter combination tested resulted in a significant decrease in cell viability compared to the negative control that did not pass a nozzle (see Figure 3). Also, no significant differences were found for optical density and urease activity after nozzle passage to the negative control (see supplement 1, 2). These results suggest that *S. pasteurii* retained its potential for MICP after passing through nozzles of sizes 150,200 and 250 μm at pressure up to 2.76 bar. The findings also indicate that there are no shear forces high enough to damage the cells and reduce their potential for MICP. Each of the parameter combinations studied is thus suitable for printing layers of biocemented sand using MICP. Further selection can therefore be based on the required flow rate and the result of uniform application of the fluids. For the proof of concept, a nozzle diameter of 200 μm and a pressure of 0.68 bar were used. This parameter combination showed subjectively the least disturbance of the sand bed when one cycle of cell suspension and calcination solution was applied.

### 3.3 Proof of concept

**FIGURE 4:**
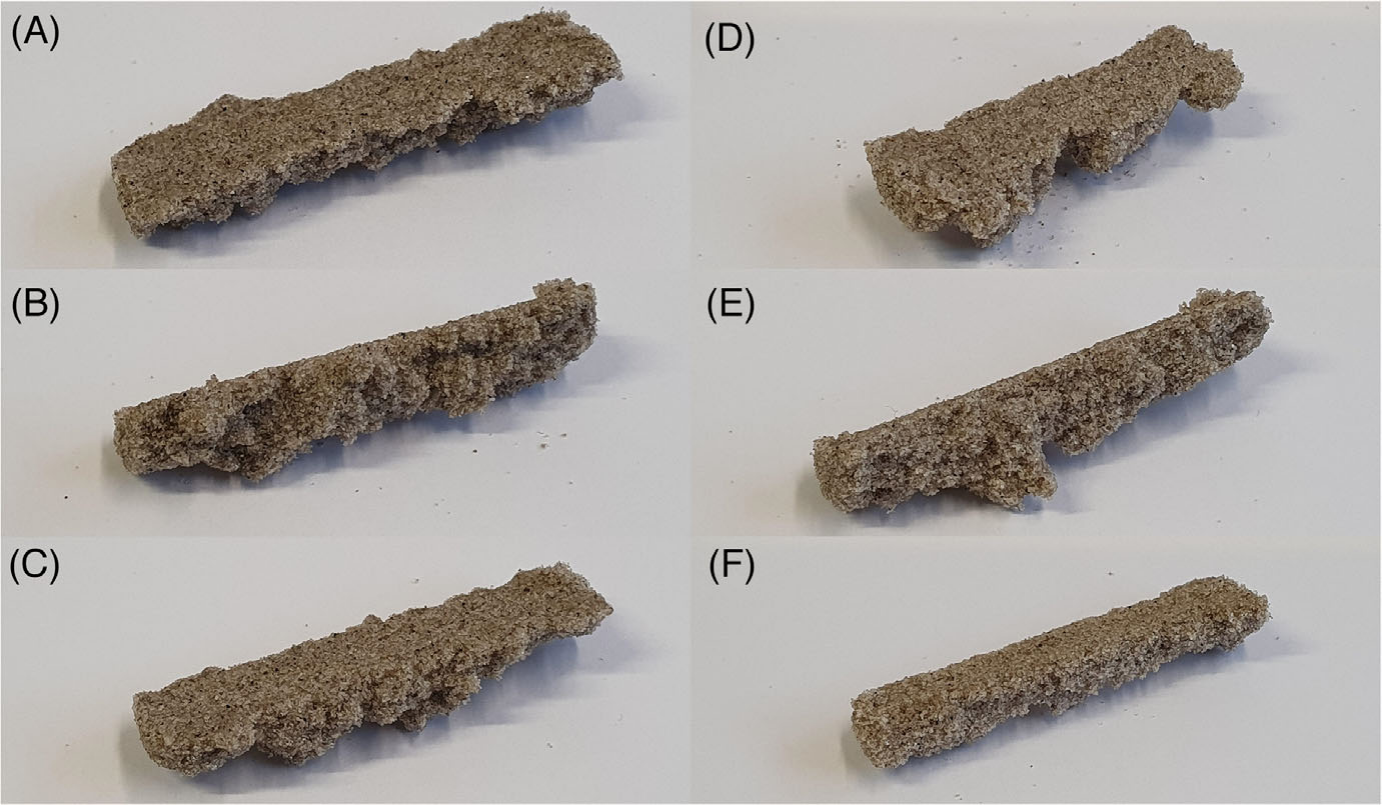
Biocemented samples after six cycles of printing with MICP. A–C) Treatment with 250 mM calcination solution D–F) Treatment with 500 mM calcination solution

**FIGURE 5:**
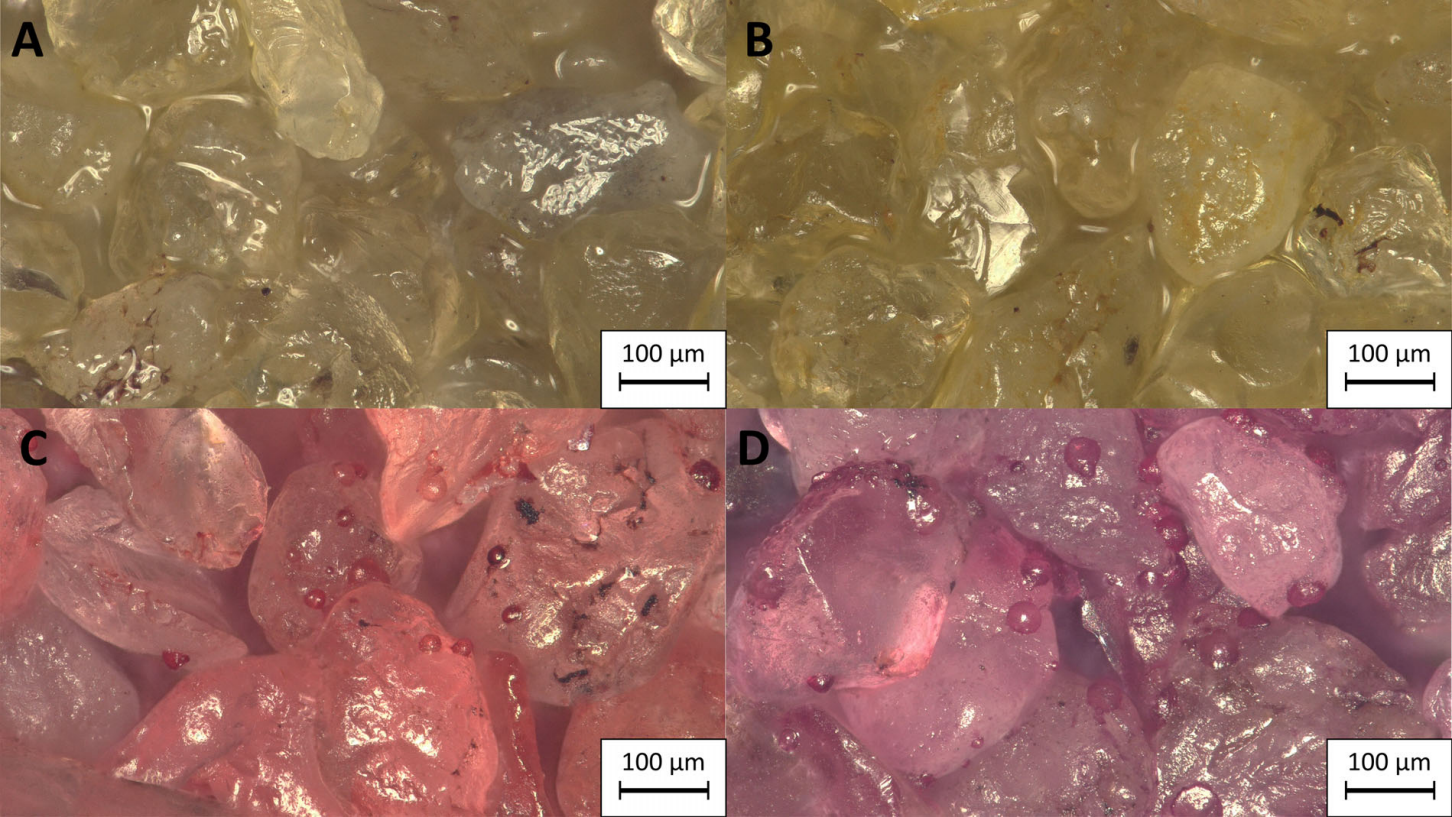
Microscopic images of the printed calcium carbonate layer on top of silica sand after six cyles of MICP and after staining with Alizarin Red S. A) Negative control, B) Treatment with cell suspension of *S. pasteurii* (OD_600_ 2.0) and 100 mM Urea/CaCl_2_, C) Treatment with (OD_600_ 2.0) and 250 mM Urea/CaCl_2_, D) Treatment with (OD_600_ 2.0) and 500 mM Urea/CaCl_2_

To demonstrate a proof of concept for printing a layer of calcium carbonate, quartz sand was placed in a container under the printer. The sand was smoothed out with a plastic card in order to obtain a homogenous surface. Now the cell suspension and the calcination solution were alternately applied by the printer. For the first tests, six printing intervals were run with a curing time of 3.5 h between each interval. Since only a limited amount of calcium carbonate can be precipitated when cells and calcination solution are added [7], up to twelve printing intervals are necessary to generate enough calcium carbonate to achieve a good detectability via microscopy . Three different equimolar concentrations of the calcination solution have been tested for printing (500, 250, and 100 mM). For concentrations of 500 and 250 mM urea and CaCl_2_ the samples were consolidated after the printing process and could be taken out of the printing bed (see Figure 4) while the samples treated with 100 mM calcination solution could not be separated from the unconsolidated sand bed without breaking apart. On microscopic examination, individual calcium carbonate crystals are visible on the sand grains, reaching a diameter of up to 26.8 respective 45.9 μm for samples treated with 250 and 500 mM calcination solution (see Figure 5). These crystals can act as bridges between individual sand grains and thus consolidate the samples. No calcium carbonate crystals were observed at a concentration of 100 mM. This is probably due to the fact that the crystals do not reach a sufficient size during a cycle of MICP and are flushed out by rinsing deionized water. Since no measurements of the compressive strength can be made due to the non‐uniform structure of the specimens, the size of the calcium carbonate crystals can give an indication of the degree of consolidation of the specimens since the compressive strength of biocemented sand increases with the size of the calcium carbonate crystals [30, 33]. The formation of the calcium carbonate crystals does not occur uniformly over the entire surface of the sand. This could be due to the fact, that the cells are flushed from the upper areas of the sand grains into depressions by the application of the cementing solution and remain there during MICP. Furthermore, cells of *S. pasteurii* agglomerate in the presence of high calcium chloride concentrations, which could lead to an uneven distribution of the cells on the sand surface. However, since the formation of bridges between particles is crucial for particle consolidation, the formation of a uniform calcium carbonate layer on the surface of the layer is not desirable [33]. So far, the possibilities of printing with MICP have been demonstrated on one layer. In further studies, the printer will be extended to print 3D objects. This will allow measurements of parameters like compressive strength and to evaluate the homogeneity of the calcium carbonate distribution in the samples in comparison to different methods of MICP. For this purpose, it is necessary to investigate under which conditions individual printed layers can be bonded together and how the cells and solutions applied to the sand are distributed in adjacent sand. Therefore, parameters such as the volume added through the nozzle during each cycle, the concentration of CaCl_2_ and urea in the calcination solution, the cell density and the number of treatments must be studied and optimized to print a 3D object. This would allow to make measurements of the compressive strength of the samples and to obtain additional data on the homogeneity in comparison to samples produced with different MICP methods.

## 4 CONCLUDING REMARKS

The potential of MICP as a technology to create sustainable construction material is limited by the necessity to create materials with a homogenous distribution of calcium carbonate. Over the last years many studies have investigated different technologies to achieve this goal. A possible solution to this problem could be 3D printing with MICP. In this study, possibilities and potential for printing calcium carbonate layers using MICP were investigated. This can be a first step to develop a 3D printing system for MICP. It was found that *S. pasteurii* can be stored at 4°C for at least 17 days after batch cultivation without major losses in optical density, ureolytic activity or cell vitality. This storage capability of the cells is important for achieving a uniform result during each treatment of MICP. Furthermore, the cells of *S. pasteurii* retain their urease activity, optical density, and cell vitality after passing through printer nozzles of various sizes (150–250 μm) and at different pressure levels (0.69–2.76 bar). That *S. pasteurii* maintains its potential of MICP after passing through the nozzles was also shown by printing a layer of calcium carbonate crystals by applying *S. pasteurii* cells and calcination solution on a bed of sand through a printer nozzle (200 μm). This technology offers the potential to enable the printing of 3D three‐dimensional objects using MICP. Further research is still necessary to develop this application of MICP into a functional technology that can be used to create homogenous construction material.

## CONFLICT OF INTEREST

The authors have declared no conflicts of interest.

## Supporting information



Supporting Information

Supporting Information

## Data Availability

The data that support the findings of this study are available from the corresponding author upon reasonable request.
