# Supplementary figures and images for: Sporosarcina pasteurii can be used to print a layer of calcium carbonate
Source: Eng Life Sci. 2022 Jun 16;22(12):760–8. doi: 10.1002/elsc.202100074 (PMC9731594; doi:10.1002/elsc.202100074)

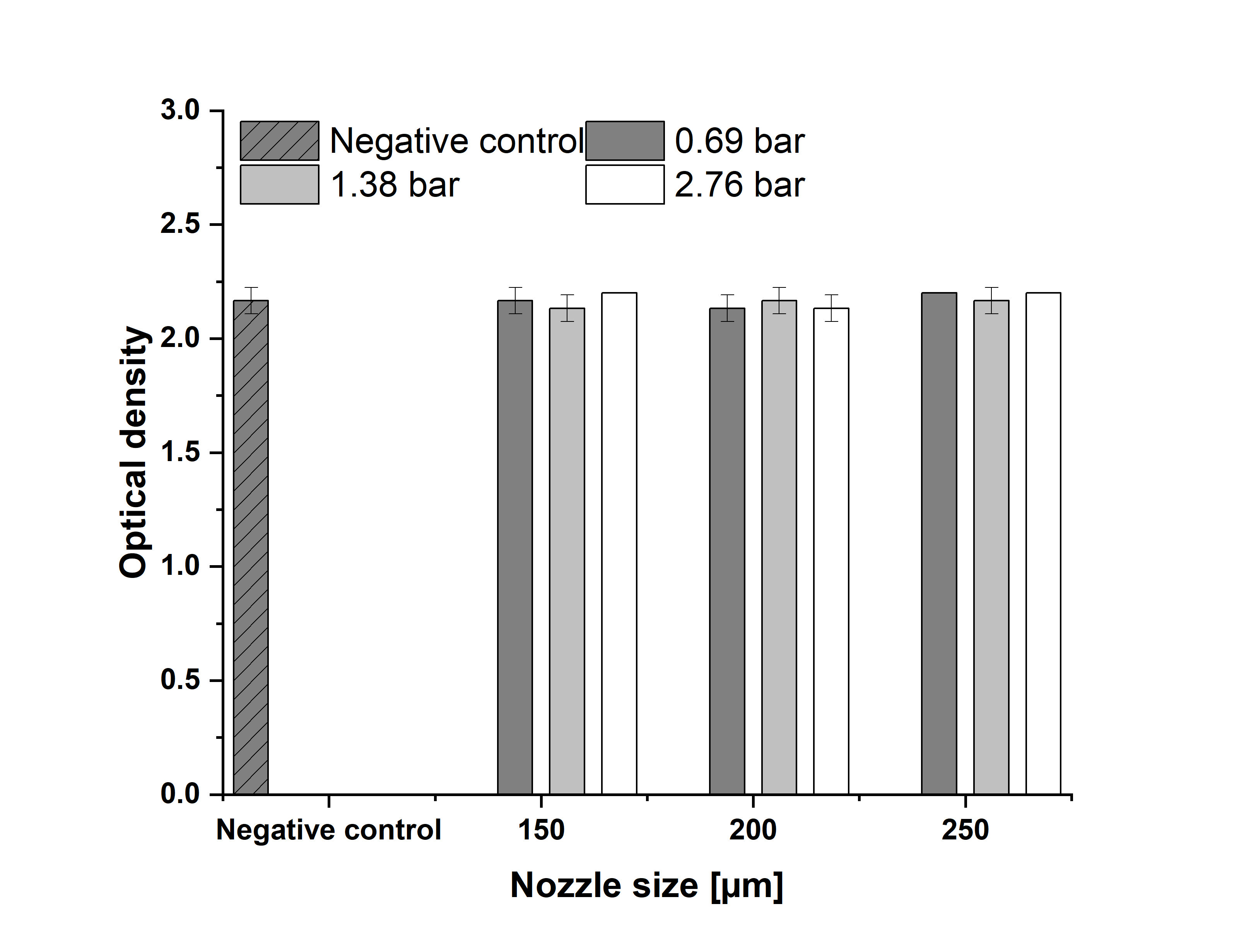

Supplement: Supplementary file 1 — Supporting Information [file ELSC-22-760-s001.tif]

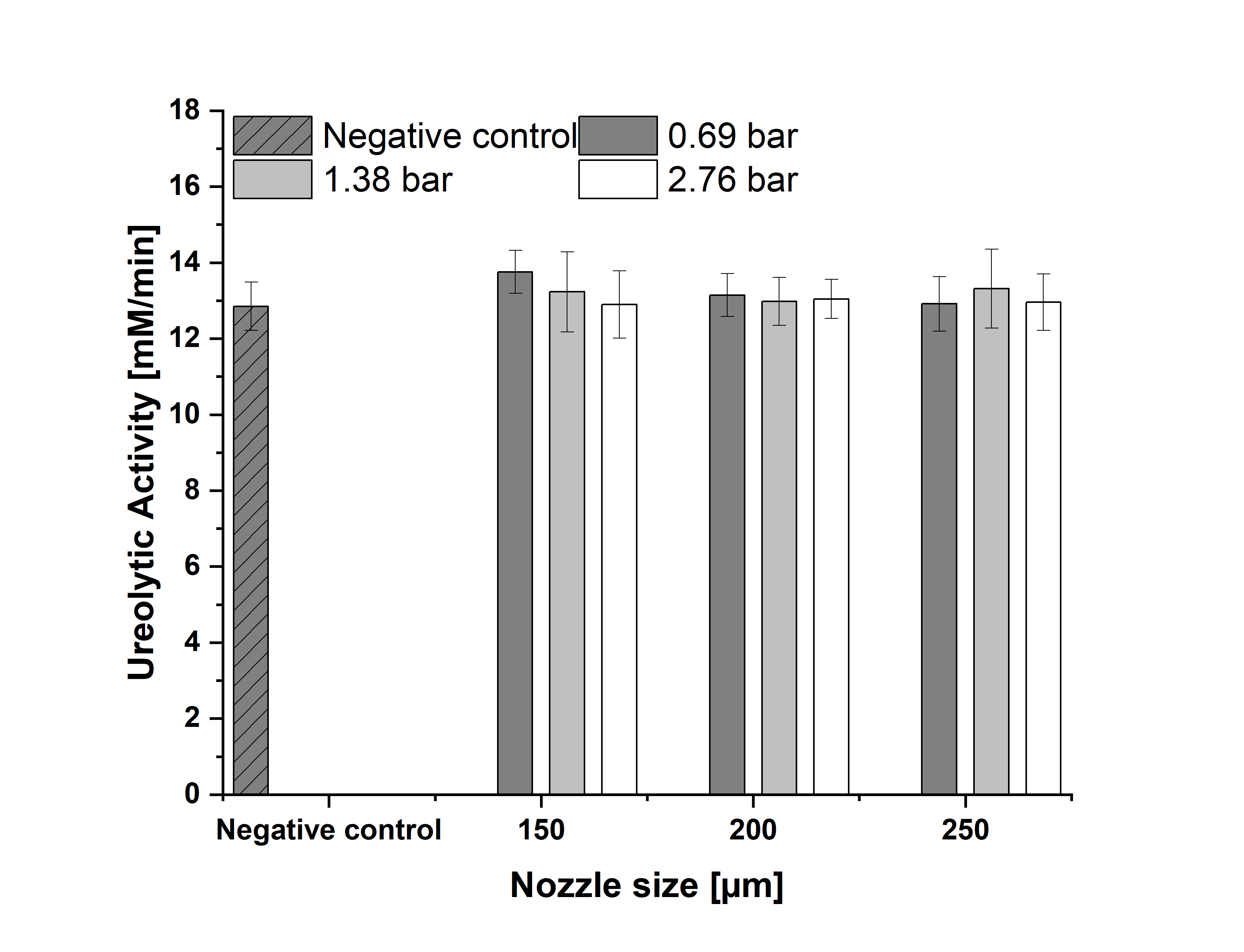

Supplement: Supplementary file 2 — Supporting Information [file ELSC-22-760-s002.tif]
